# Prevalence of Celiac Disease in Patients With Primary Biliary Cholangitis: A Systematic Review and Meta‐Analysis

**DOI:** 10.1111/liv.70293

**Published:** 2025-08-19

**Authors:** Fabiana Zingone, Cristina Canova, Anders Forss, Fahim Ebrahimi, Nora Cazzagon, Isabella Rosato, Jonas F. Ludvigsson

**Affiliations:** ^1^ Department of Surgery, Oncology and Gastroenterology University of Padua Padua Italy; ^2^ Gastroenterology Unit Azienda Ospedale‐Università Padova Padua Italy; ^3^ Unit of Biostatistics, Epidemiology and Public Health (UBEP), Department of Cardiac, Thoracic, Vascular Sciences and Public Health University of Padua Padua Italy; ^4^ Department of Medical Epidemiology and Biostatistics Karolinska Institutet Stockholm Sweden; ^5^ Centre for Digestive Health, Department of Gastroenterology, Dermatovenereology and Rheumatology Karolinska University Hospital Stockholm Sweden; ^6^ Department of Gastroenterology and Hepatology University Digestive Health Care Center Basel – Clarunis Basel Switzerland; ^7^ Department of Paediatrics Örebro University Hospital Örebro Sweden; ^8^ Department of Medicine Columbia University College of Physicians and Surgeons New York USA

**Keywords:** coeliac disease, prevalence, primary biliary cholangitis, systematic review

## Abstract

Celiac disease (CeD) has been linked to both autoimmunity and chronic liver disease, but most data on the link to primary biliary cholangitis (PBC) originate from small studies and have yielded conflicting results. A systematic search was performed in the databases of Medline, Embase, Cochrane and Web of Science Core Collection for studies published between 1990 and 2024, using search terms related to CeD, gluten and PBC. The search identified 2016 publications, of which 94 were read in full text. Of these, a total of 25 studies were included in this review, with 22 deemed relevant for meta‐analysis. We applied a random effects model to estimate the weighted pooled prevalence along with corresponding 95% confidence intervals (CIs). Results were reported in accordance with the PRISMA guidelines. Our main analysis contained 15 006 individuals with PBC from 22 studies. Among these, 286 (1.9%; 286/15174) had a CeD diagnosis yielding a pooled prevalence of 1.71% (95% CI: 1.08–2.44). When restricting the analysis to 15 studies with biopsy‐confirmed CeD, the pooled prevalence was 1.53% (95% CI: 0.51–2.91). This prevalence is comparable to the serology‐based prevalence of CeD, of around 1%, in the general population. Our findings suggest that the prevalence of CeD in PBC is similar to that observed in the general population. These findings do not support routine screening for CeD in individuals with PBC.

**Summary**

- Primary biliary cholangitis (PBC) is a chronic autoimmune liver disease that can progress to cirrhosis if untreated. It is commonly associated with other autoimmune diseases.
- Celiac disease (CeD) is an autoimmune disorder triggered by gluten ingestion, leading to intestinal damage. Some guidelines have suggested screening for CeD in PBC patients, but evidence remains unclear.
- This systematic review and meta‐analysis included over 15 000 PBC patients, estimating a 1.71% pooled prevalence of CeD.
- The prevalence of CeD in PBC patients was similar to that of the general population (1%), suggesting that routine CeD screening in PBC patients may not be necessary unless clinically indicated.
- The study provides important clinical insights on the coexistence of PBC and CeD and challenges the need for universal screening of CeD in PBC patients.

**Abbreviations**
AASLD: American Association for the Study of Liver Diseases
ALP: alkaline phosphatase
AMA: antimitochondrial antibodies
ANA: antinuclear antibodies
ASReview: active learning software for systematic reviews
CeD: celiac disease
CI: confidence interval
EASL: European Association for the Study of the Liver
EMA: Endomysial antibodies
ICD: International Classification of Diseases
JBI: Joanna Briggs Institute
PBC: primary biliary cholangitis
PRISMA: preferred reporting items for systematic reviews and meta‐analyses
PROSPERO: International prospective register of systematic reviews
STATA: Statistical software package
tTG: tissue transglutaminase
UEG: United European gastroenterology

## 1 Introduction

Primary biliary cholangitis (PBC) is a slowly progressive autoimmune cholestatic disorder that can lead to cirrhosis and liver failure requiring liver transplant if left untreated or diagnosed late [1]. PBC predominantly affects females (female‐to‐male ratio 4:1) [2] and is frequently associated with other autoimmune diseases such as Hashimoto's thyroiditis and rheumatoid arthritis [3]. The pooled global prevalence of PBC is estimated at 14.60 per 100 000 person/years with significant variations between studies [4, 5, 6]. Clinical manifestations of PBC include fatigue, pruritus, and right upper quadrant abdominal discomfort. The diagnosis of PBC is confirmed through a combination of the following criteria: [1] persistent elevation of cholestatic liver enzymes, including alkaline phosphatase (ALP); [2] detection of antimitochondrial antibodies (AMA) or PBC‐specific anti‐nuclear (ANA) autoantibodies (anti‐sp100 or anti‐gp210); and/or [3] typical histopathology features at liver biopsy [7].

Celiac disease (CeD) is an autoimmune disorder affecting the small bowel and triggered by ingestion of gluten in genetically predisposed individuals [8]. Globally, the seroprevalence of CeD is about 1.4%, while the prevalence of biopsy‐confirmed CeD is 0.7% [9]. Compatible serologic testing and duodenal biopsies showing villous atrophy are the key diagnostic criteria for CeD. Similar to PBC, CeD is often associated with other autoimmune disorders such as Hashimoto's thyroiditis and type 1 diabetes [10].

To date, data on the prevalence of CeD in PBC have been contradictory [11, 12, 13, 14, 15, 16, 17]; the true prevalence of CeD is unknown. In this study, we aimed to estimate the pooled prevalence of CeD in PBC through a systematic review and meta‐analysis of the existing literature.

## 2 Methods

This review was reported in accordance with the preferred reporting items for systematic reviews and meta‐analyses (PRISMA) guidelines [18]. A prespecified protocol was registered in the PROSPERO database (Protocol ID: CRD42024568767).

### 2.1 Search

This literature review was carried out by a librarian at the library of Karolinska Institutet Library (Stockholm, Sweden) (Table S1), and was conducted through Medline, Embase, Cochrane and Web of Science Core Collection databases from January 1, 1990 to March 12, 2024 including the terms ‘primary biliary cholangitis’ and ‘liver cirrhosis’, combined with ‘celiac disease’, ‘non‐tropical sprue’, ‘glutens’, ‘transglutaminases’, ‘endomysium’ and ‘villous atrophy’. Search strategies were elaborated together with an experienced librarian at the library of Karolinska Institutet Library (Stockholm, Sweden) (Table S1).

We included all studies providing information on the prevalence of CeD in patients with a diagnosis of PBC. We defined a CeD diagnosis as the presence of a positive small intestinal biopsy or a CeD diagnosis retrieved from clinical local records or national/international databases, assuming that these diagnoses adhered to CeD guidelines [19]. When CeD was reported as ‘biopsy‐verified CeD’ or similarly, without explicit reference to Marsh stages, we assumed the biopsy corresponded to Marsh stages II or III. A sensitivity analysis, excluding patients whose diagnoses were obtained from databases, was performed.

Moreover, as a secondary analysis, we investigated the prevalence of positive CeD serology markers (when available) in PBC. In particular, we separately considered the prevalence when tissue transglutaminase (tTG) IgA antibodies were positive and when both tTG IgA and IgA EMA were positive.

The definition of PBC was based on the European Association for the Study of the Liver (EASL) 2017 [6] and/or the American Association for the Study of Liver Diseases (AASLD) 2018 [7] criteria by meeting at least 2 of the 3 criteria (A–C): (A) Biochemical evidence of cholestasis, defined as elevated ALP. (B) Presence of anti‐mitochondrial antibody (AMA) at a titre > 1:40 or PBC‐specific antinuclear antibodies (ANA) in immunofluorescence (nuclear dots or perinuclear rims) or positive ELISA results (sp100, gp210). (C) Histologic evidence of non‐suppurative destructive cholangitis and destruction of interlobular bile ducts *or* an ICD code for PBC (ICD‐9: 571G; ICD‐10: K74.3).

We included cross‐sectional studies, longitudinal studies (observational or clinical), randomised controlled trials, as well as register‐based studies and patient chart reviews. We limited the search according to English language, while we did not limit to sample size, age group, sex, or country/region. Case reports, commentaries, and conference abstracts were excluded.

The title and abstract screening was performed using ASReview version 1.3 [20], a software that uses artificial intelligence to speed up the title/abstract screening phase. ASReview employs active learning to prioritise articles based on their relevance to the inclusion process. A single reviewer (F.Z.) provided the initial training data to ASReview, and the classifier created a progressive ranking of the unseen records. The same reviewer then screened the ranked papers for relevance. When many articles are excluded consecutively, it can be assumed that the subsequent articles can be labelled as irrelevant. The software developers advise a screen‐stop decision after 100–120 consecutively excluded studies [21]. To ensure we would not miss relevant studies, we opted to stop screening after 270 consecutive excluded studies.

The full‐text screening was then performed using Covidence software (Covidence systematic review software, Veritas Health Innovation, Melbourne, Australia. Available at www.covidence.org.) by two independent reviewers (C.C. and F.Z.), considering all the papers included after the initial screening in ASReview for final inclusion in the review. In cases of disagreement on inclusion/exclusion, screening, review, or interpretation of the studies, the issue was resolved through discussion between the two independent reviewers, and no disagreement remained. Additionally, reference lists of the included papers were screened to search for potentially relevant studies not captured by the search strategies.

### 2.2 Data Extraction and Risk of Bias

Two independent reviewers (F.Z. and C.C.) systematically extracted data on the following variables into a study‐specific extraction form (in Microsoft Excel): (1) first author and year of publication, (2) country and region where the study was conducted, (3) sample size (including proportion of males/females) and mean/median age, (4) number of CeD cases, (5) Marsh stage/villous atrophy, (6) diagnostic criteria for PBC, (7) serological markers of CeD and (8) number of cases with positive CeD serology (tTG IgA positive and both tTG IgA + EMA positive, separately) (Table 1). The Joanna Briggs Institute/JBI Critical Appraisal Checklist for included Prevalence Studies [45] was used to grade the quality of included studies (Table S2).

**TABLE 1:** Publications included in the systematic review on coeliac disease in PBC (*N* = 25).

Study	Country	Patients with PBC, *n*	Female (%)	Age (mean)	IgA tTG positive	IgA EMA positive, *n*	AGA positive, *n*		IgA tTG + EMA positive, *n*	Biopsy confirmed CeD, *n*	Proportion of patients that underwent biopsy, *n*	Diagnostic criteria
							IgG	IgA				
Sjoberg, 1997 [22]	Sweden	101	91	63	NA	0	4	16	NA	0	0/0	Biopsy if positive serology (EMA if IgA AGA/IgG AGA pos)
Dickey, 1997 [23]	Northern Ireland	57	91	57	NA	6	NA	NA	NA	4	4/6	Biopsy if positive serology (EMA)
Fidler, 1998 *(letter to the editor)* [24]	UK	87	NA	NA	NA	2	NA	NA	NA	2	2/2	Biopsy if positive serology (EMA)
Volta, 1998 [25]	Italy	62	94	59	NA	0	11	0	NA	0	0	Biopsy if positive serology (EMA)
Kingham, 1998 [12]	UK	67	NA	NA	NA	NA	NA	NA	NA	4^a^	NA	Clinical National Register from a stable population in South Wales
Gillett, 2000 [26]	Canada	378	90	53.3	54	10	NA	NA	10	5	5/10	Biopsy if positive serology (tTG and EMA)
Volta, 2002 [27]	Italy—Spain	173	90	56	7	7	NA	3	7	7	7/7	Biopsy if positive serology (EMA + tTG IgA)
Chatzicostas, 2002 [14]	Greece	62	85	59	6	0	3	12	0	0	10/17	Biopsy if positive serology (at least one of: AGA/EMA/tTG)
Kaukinen, 2002 [28]	Finland	61	NA	NA	5	3	NA	NA	3	3	5/5	Biopsy if positive serology (tTG). Diagnosis before liver transplantation (OLT).
Floreani, 2002 [29]	Italy	87	91	55	24	3	NA	NA	3	3	20/24	Biopsy if positive serology (tTG)
Bizzaro, 2003 [30]	Italy	48	NA	NA	4	0	NA	NA	0	0	0	Biopsy if positive serology (EMA + tTG)
Habior, 2003 [31]	Poland	115	94	55^d^	7	1	NA	1	0	1	5/8	Biopsy if positive serology (tTG or EMA)
Germenis, 2005^b^ [32]	Greece	48	85	59	8	0	NA	NA	0	NA	Unknown	Biopsy if positive serology (tTG)
Bizzaro, 2006 [33]	Italy	105	87	63	28^c^	2	NA	NA	2	2	6/7	Biopsy if positive serology (tTG + HLA pos or if tTG + EMA pos)
Rubio‐Tapia, 2008^b^ [34]	USA	112	NA	NA	7 (1 HLA neg)	2	NA	NA	2	NA	Unknown	Positive serology (EMA + tTG) – Data available pre and post liver transplantation (OLT). Post OLT *EMA normalised, unclear for tTG IgA*
Biagini, 2008 [35]	Italy	44	90	58.9	NA	NA	NA	NA	NA	1	NA	Clinical records from a single centre
Mirzaagha, 2010^b^ [36]	Iran	8	100	50.2	1	NA	NA	NA	NA	NA	0/1	Biopsy if positive serology (tTG)
Gatselis, 2012 [37]	Greece	45	84	59.1	7	NA	NA	3 (DGP IgA)	NA	0	2/7	Biopsy if positive serology (IgG/IgA DGP or/and IgA tTG)
Wakim Fleming, 2014 [38]	USA	12	NA	NA	0	0	NA	NA	0	0	0	Biopsy if positive serology (tTG + EMA)
Floreani, 2015 [39]	Italy	361	94	53.1	NA	NA	NA	NA	NA	5		Clinical records from a single centre database
Muratori, 2015 [40]	Italy	281	89	53^d^	NA	NA	NA	NA	NA	14		Clinical records from a single centre database
Callichurn, 2021 [41]	Canada	51	46	NA	6	NA	NA	NA	NA	6	6/6	Biopsy if positive serology (tTG)
Efe, 2021 [42]	Turkey	1554	87	54.1	NA	NA	NA	NA	NA	26		Clinical records from databases from 20 different centres
Yehezkel, 2021 [43]	Israel	125	93	54.7	NA	NA	NA	NA	NA	3		Clinical records from single centre database
Hitawala, 2023 [44]	USA	11 130	85	NA	115	NA	NA	NA	NA	200		Clinical records from a multicentre validated database

Abbreviations: NA: not available; PBC, Primary biliary cholangitis.

^a^ 2/67 patients with PBC were diagnosed with CeD after PBC diagnosis; two already had a diagnosis of CeD.

^b^ Excluded from the main meta‐analysis because of unclear outcome.

^c^ The HLA haplotype was determined in 24 of the 26 anti‐tTG‐positive and EMA‐negative patients, as two of them were dead. Five of the 24 were HLA positive and four underwent a duodenal biopsy.

^d^ only medians were reported (the two studies were not included in the meta‐regression analysis by mean).

### 2.3 Statistics

We used the ‘metaprop’ STATA (STATA/BE 18.0, College Station, TX, USA) command [46] for performing meta‐analysis of binomial data. We choose ‘metaprop’ because it implements specific procedures for binomial data and is appropriate for dealing with proportions close to or at the margins (0% and 100%). It also uses the Freeman‐Tukey Double Arcsine Transformation to stabilise the variances. The heterogeneity between studies was estimated through the *I* ^2^ statistics. We defined an *I* ^2^ > 50% as indicating substantial heterogeneity. We expected a high degree of heterogeneity among the studies of this meta‐analysis and therefore used a random effects model to calculate the weighted pooled prevalence along with their 95% confidence intervals (CIs). Potential publication bias was examined through funnel plots and by applying the Egger's test.

We conducted subgroup analyses stratified by region Asia (Iran, Turkey and Israel) vs. Europe (Italy, Sweden, Northern Ireland, UK, Spain, Greece, Finland and Poland) vs. North America (USA and Canada). Through meta‐regression, we also examined the association of CeD prevalence in PBC with publication year, mean age of the study population, proportion of females, and sample size. These study characteristics were chosen based on the assumption that they could explain the potential variance of CeD prevalence between studies.

STATA version 18 (STATA/BE 18.0, College Station, Texas) was used for all analyses. Microsoft Excel (v. 16.54, 2021, Microsoft Corporation, Washington, USA) was used for compiling and managing extracted data.

### 2.4 Ethics

This was a study of the existing literature and hence no ethical permission was needed.

## 3 Results

**FIGURE 1:**
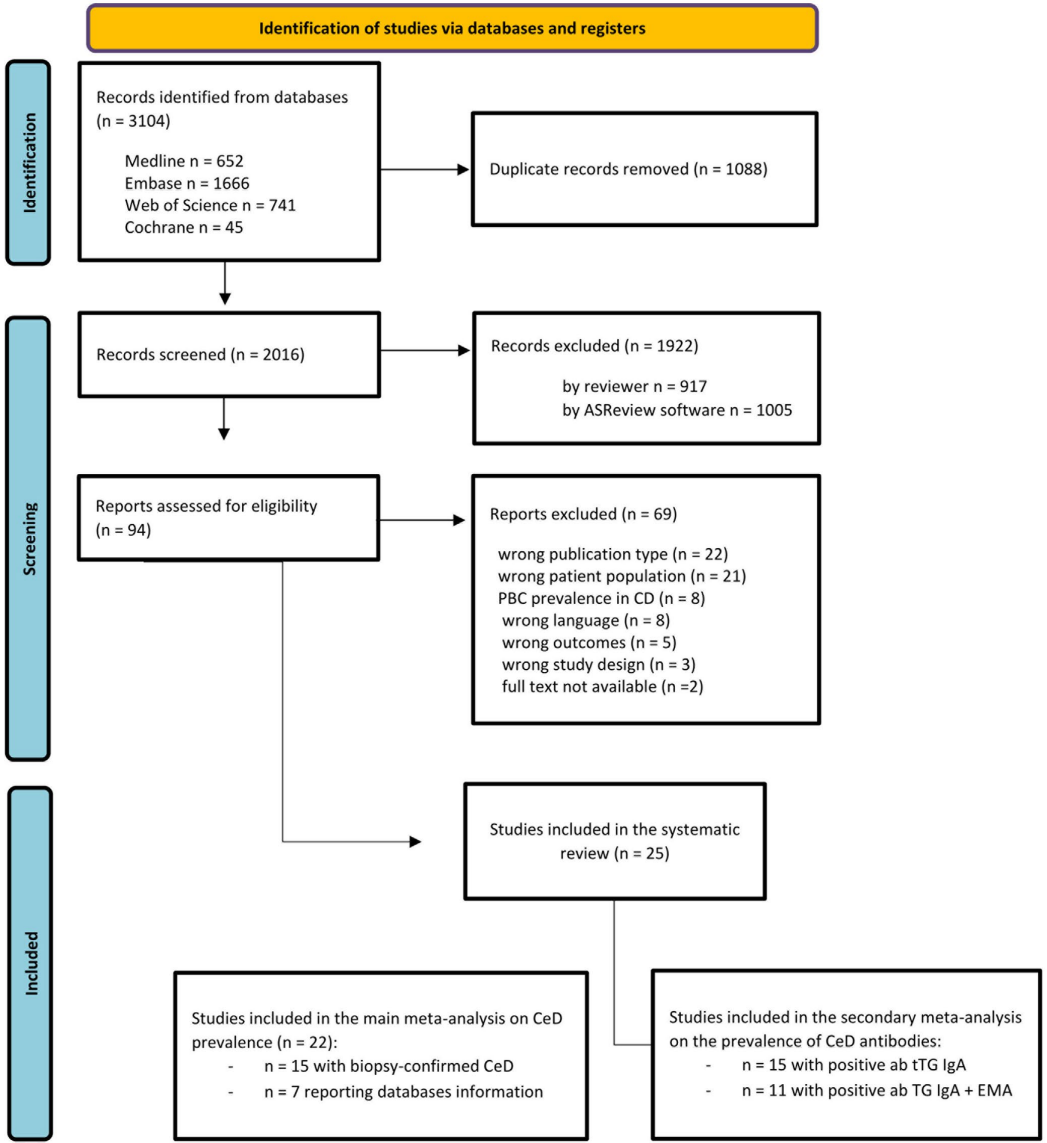
PRISMA flowchart.

A total of 2016 titles and abstracts from publications published between January 1, 1990 and March 12, 2024 were screened. Using the ASReview tool, 45.2% of these publications were classified as irrelevant. A total of 94 publications were identified as potentially relevant for inclusion and read in full. Of these, 69 were excluded for the following reasons: wrong publication type (e.g., letters to the editors or conference abstracts) (*n* = 22), irrelevant patient population (*n* = 21), studies about the PBC prevalence in celiac patients (*n* = 8), not in English (*n* = 8), wrong outcome (*n* = 5), wrong study design (*n* = 3), full text not available (*n* = 2). The remaining 25 publications [12, 14, 22, 23, 24, 25, 26, 27, 28, 29, 30, 31, 32, 33, 34, 35, 36, 37, 38, 39, 40, 41, 42, 43, 44] were included in the systematic review. Of these, 22 were included in the main meta‐analysis since in three studies [32, 34, 36] the number of patients who underwent biopsies among those with positive serology results was not reported, or duodenal biopsies were not performed at all. The PRISMA flowchart is presented in Figure 1.

Study characteristics of all included publications are reported in Table 1. Quality assessment of included studies was conducted using the Joanna Briggs Institute (JBI) Critical Appraisal Checklist for prevalence studies (Table S2).

### 3.1 Prevalence of Coeliac Disease in PBC

In the main meta‐analysis, we included 22 studies [12, 14, 22, 23, 24, 25, 26, 27, 28, 29, 30, 31, 33, 35, 37, 38, 39, 40, 41, 42, 43, 44], comprising a total of 15 006 individuals (85% female), with a mean age of 56 years at the time of the study. Of these studies, 15 reported biopsy‐confirmed CeD, while in seven studies, individuals with CeD were identified through information provided by clinical records from single or national and international registers (Table 1).

**FIGURE 2:**
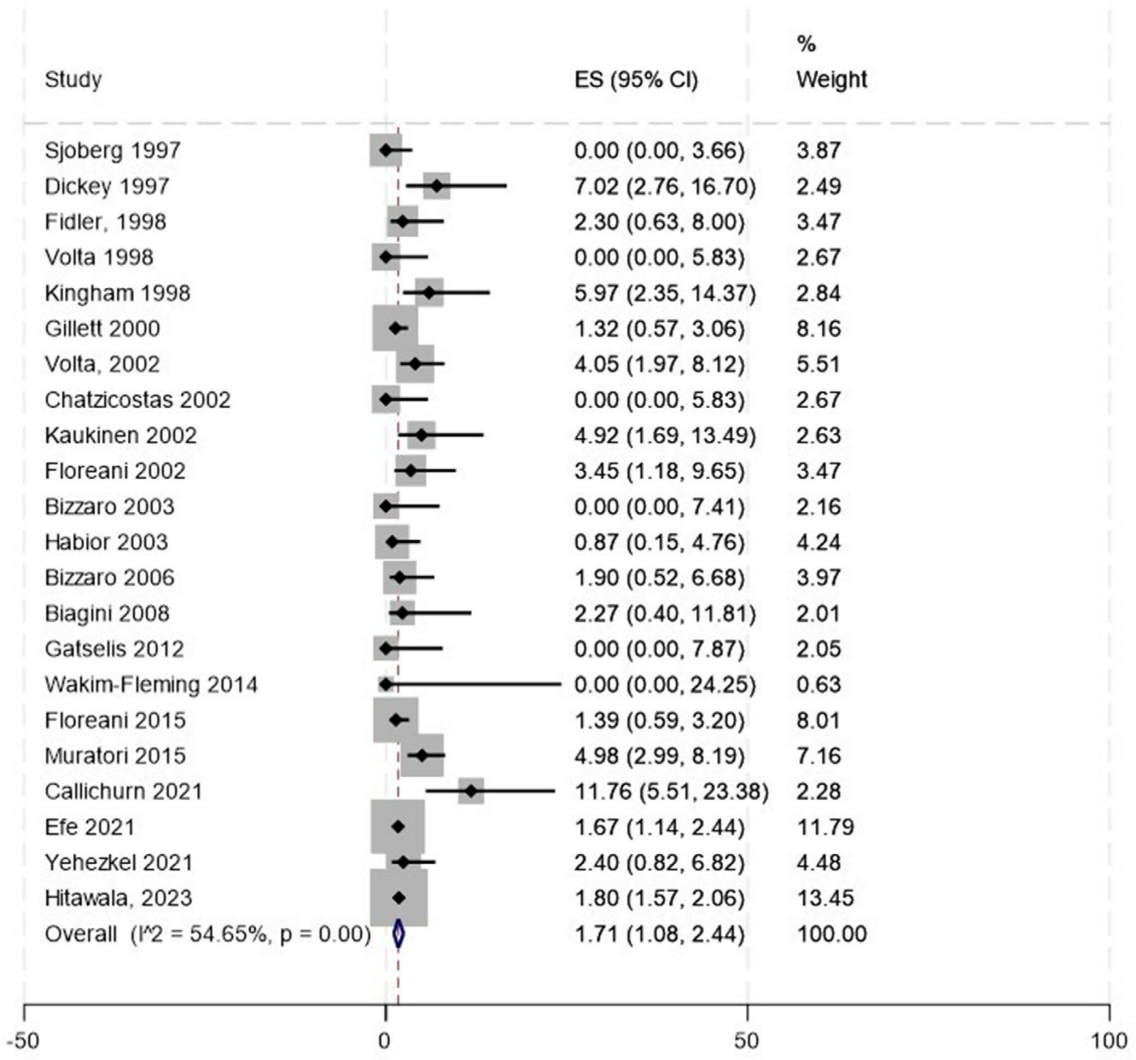
Prevalence of coeliac disease diagnosis in PBC (*n* = 22).

Across all 22 studies, we found 286 patients with CeD among 15 006 PBC patients, with a crude prevalence of 1.9%. The weighted pooled prevalence of CeD in patients with PBC was 1.71% (95% CI: 1.08–2.44), with an *I* ^2^ of 54.65% (Figure 2).

Due to concerns that the studies by Floreani et al., published in 2002 [29] and 2015 [39], overlapped, we performed a sensitivity analysis excluding the older study [29]. This yielded a weighted pooled prevalence of CeD of 1.65% (95% CI: 1.03–2.39).

**FIGURE 3:**
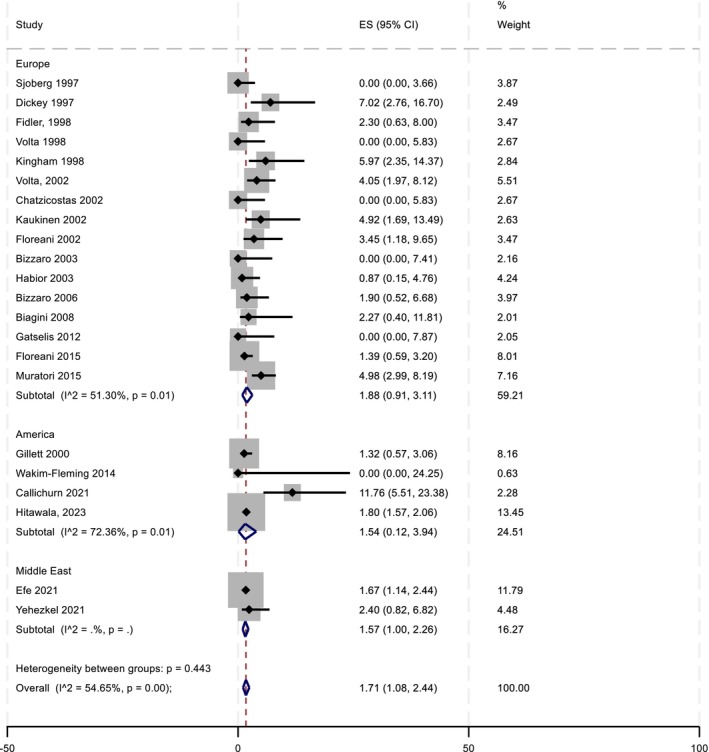
Prevalence of coeliac disease diagnosis in PBC by geographical region (*n* = 22).

When considering the geographical regions where the studies were conducted, those from Europe (*n* = 16) showed a weighted pooled prevalence estimate of 1.88% (95% CI 0.91–3.11), which was slightly higher than the estimates from studies conducted in North America (*n* = 4) at 1.54% (95% CI 0.12–3.94) and in the Middle East (*n* = 2) at 1.57% (1.00–2.26) (Figure 3).

Moreover, restricting the main analysis to the 15 studies with biopsy‐confirmed CeD [14, 22, 23, 24, 25, 26, 27, 28, 29, 30, 31, 33, 37, 38, 41], excluding studies reporting CeD diagnosis retrieved from databases, resulted in a prevalence of 1.53% (95% CI: 0.51–2.91; *I* ^2^ = 54.27%).

Meta‐regression analyses assessing the association between CeD prevalence in PBC and factors such as publication year (*p* = 0.558), sample size (*p* = 0.734), proportion of females (*p* = 0.693), and mean age (*p* = 0.323) revealed no significant associations (Figures S1–S4).

**FIGURE 4:**
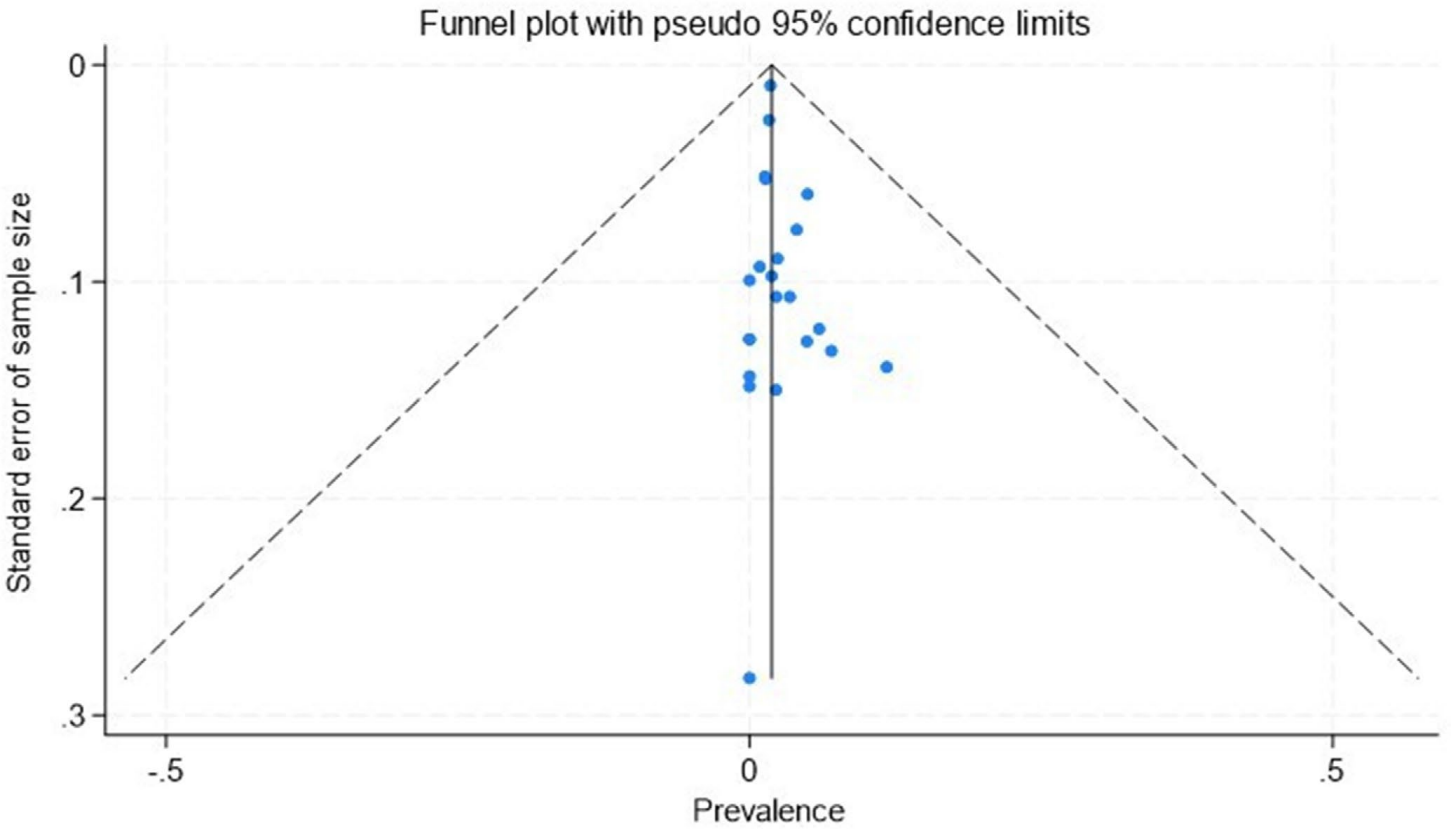
Funnel plot of studies investigating biopsy‐confirmed coeliac disease in patients with PBC.

No major publication bias was detected based on the funnel plot (Figure 4) or Egger's test (PBC, *p* = 0.157).

### 3.2 Prevalence of Coeliac Disease Antibodies in Patients With PBC

**FIGURE 5:**
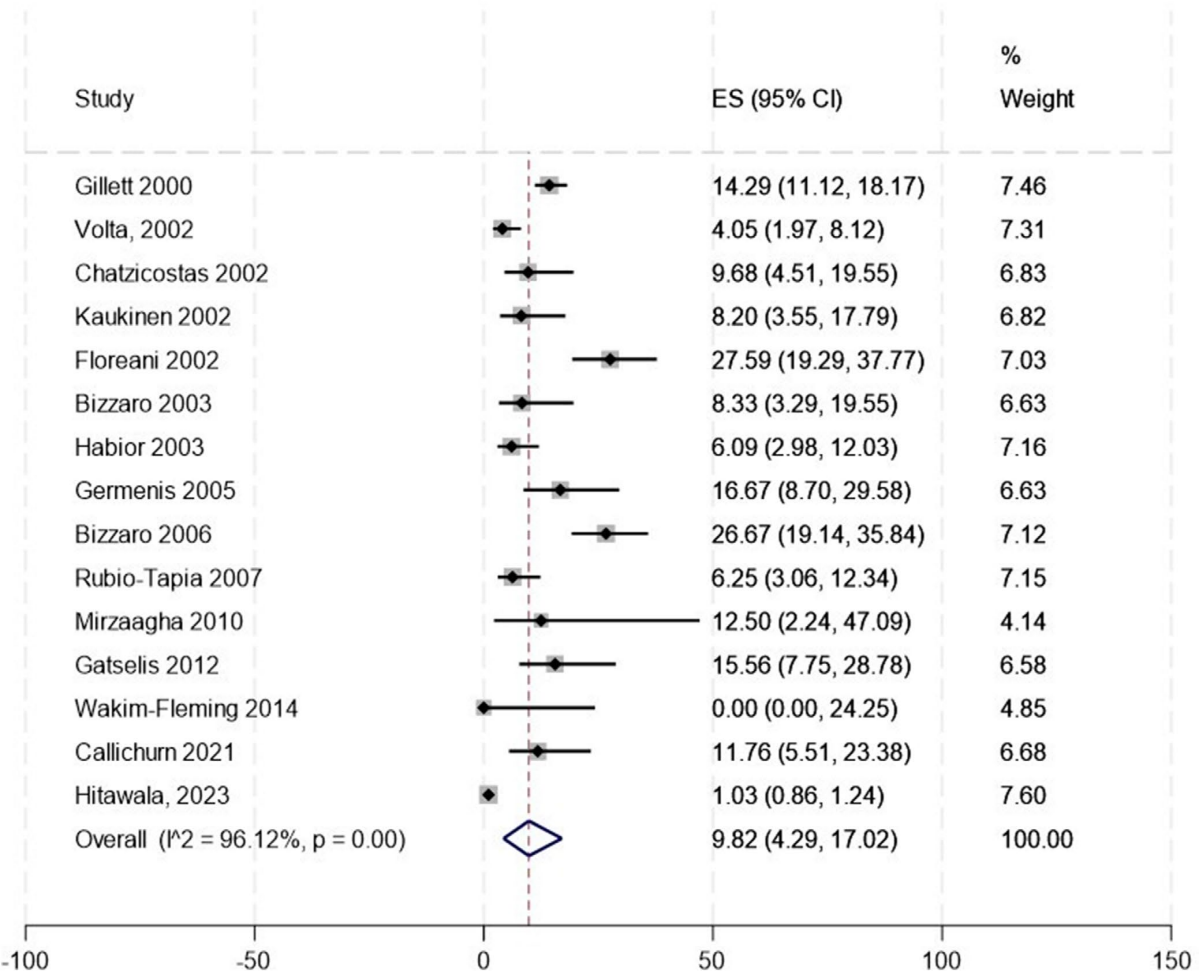
Prevalence of anti‐transglutaminase IgA positivity in PBC (*n* = 15).

Conversely, in a secondary meta‐analysis including all studies in which patients had at least positive IgA tTG, even in the absence of EMA positivity or biopsy‐confirmed CeD (*n* = 15) [14, 26, 27, 28, 29, 30, 31, 32, 33, 34, 36, 37, 38, 41, 44], we found the highest weighted pooled CeD prevalence at 9.82% (95% CI 4.29–17.02) (Figure 5). After excluding the two studies with the highest reported CeD prevalence (Floreani A et al., 2002 (27.6%) and Bizzaro N et al., 2006 (26.7%)) [29, 33], the prevalence decreased to 7.53% (95% CI 2.96–13.64).

Additionally, we performed a meta‐analysis on the prevalence of positive serological CeD markers (IgA EMA plus IgA tTG antibodies) in PBC (*n* = 11 studies) [14, 26, 27, 28, 29, 30, 31, 32, 33, 34, 38], observing a weighted pooled CeD prevalence of 1.32% (95% CI 0.45–2.51), which was similar to the biopsy‐confirmed prevalence (Figure S5).

## 4 Discussion

In this systematic review and meta‐analysis of 15 006 patients with PBC, the pooled weighted prevalence of biopsy‐confirmed CeD was 1.71%. To our knowledge, this is the first meta‐analysis to report the prevalence of CeD in patients with PBC. Notably, the estimated CeD prevalence of 1.71% in PBC patients was comparable to that of the general adult population (1%) [9]. When the analysis was restricted to individuals with double positive serological CeD tests (EMA and tTG IgA), the prevalence was even lower (1.34%). These results do not support routine CeD screening in PBC patients.

Both PBC and CeD can present with elevated transaminase levels at diagnosis, making it essential to consider both conditions in the evaluation of hypertransaminasemia. Additionally, PBC and CeD share certain clinical features, such as osteoporosis and fatigue, which can be particularly debilitating when the two conditions coexist. Furthermore, the presence of duodenal atrophy may impair drug absorption, leading to an incomplete therapeutic response to drugs used in PBC. Current expert recommendations [10, 11] advise screening for CeD in patients diagnosed with PBC. However, based on our findings, routine screening for PBC in patients with CeD is not warranted unless there is a specific clinical suspicion of PBC.

It is important to highlight the high prevalence of tTG IgA positivity found in this meta‐analysis, suggesting the possibility of false‐positive tTG IgA results in patients with chronic liver diseases [47, 48]. The lower sensitivity of anti‐tTG might be linked to the presence of low‐titre positive IgA anti‐tTG antibodies, which can also be detected in other conditions such as hypergammaglobulinaemia, autoimmune diseases, congestive heart failure, and gastroenteritis [49]. This underscores the need for confirmatory testing before diagnosing CeD, particularly in this clinical context. This information is especially relevant in the era of a no‐biopsy approach to CeD diagnosis [50, 51]. Unfortunately, most studies lack information regarding tTG levels, particularly in cases of false‐positive results, and do not consistently report the specific test methods used. Notably, as early as 2003, Bizzaro et al. [30] analysed potential causes of tTG IgA positivity, distinguishing between true CeD, a future CeD, true positivity due to autoimmune diseases with marked B‐polyclonal activation, and false positivity associated with other conditions.

PBC and CeD differ in terms of age and sex distribution. PBC exhibits a marked female predominance, with an approximately 4:1 ratio, while CeD shows a less pronounced female predominance. The mean age of onset for PBC typically falls in middle age, with most diagnoses occurring between 40 and 60 years. On the other hand, CeD can manifest at any age, from infancy to late adulthood, with two diagnostic peaks: one in early childhood and another in the third to fourth decades of life.

To explore potential associations between patient and study characteristics and CeD prevalence, we performed a series of meta‐regression analyses. No significant associations were found between CeD prevalence and factors such as publication year, mean age of the study population, or sex, suggesting that these features likely do not influence the outcome.

Our study results contrast those of an earlier Swedish population‐based study [16]. In that study, patients with CeD were at a 10‐fold increased risk of having a later PBC diagnosis [16]. However, CeD patients are often regularly tested for liver disease as opposed to the general population where increased surveillance and awareness of PBC probably played a role in that study. That study also used a general population as comparators, which are generally not screened for liver disease.

The strengths of the current study include the large number of identified studies drawn from four major databases (Medline, Embase, Cochrane and Web of Science Core Collection). The relatively large number of included studies enabled us to restrict the analysis to cases with histologically confirmed CeD (then with a pooled prevalence of 1.53%), rather than relying solely on serological markers. This is particularly relevant, as a diagnosis based only on serology may overestimate the true prevalence of CeD, especially when relying on a single test.

We acknowledge some limitations to our findings. First, we lack data from certain geographic regions, such as Sub‐Saharan Africa and South America, but also information on ethnicity. Second, in some of the studies included in our meta‐analysis, not all patients with elevated levels of CeD serological markers underwent biopsy. Third, the criteria used for performing duodenal biopsies were not standardised across studies: in some studies, all patients with positive tTG IgA underwent upper endoscopy with biopsy, while in other studies only patients with double‐positive serology (EMA and tTG) were biopsied. We cannot rule out that this variability may have underestimated the true prevalence of CeD. However, this concern is mitigated by our finding that even when considering only double‐positive cases, CeD prevalence remained below 2% (approximately 1.3%). Fourth, the lack of data on the immunosuppressive therapies of patients is a limitation that should be considered when interpreting the results. This issue is emphasised in the study by Rubio‐Tapia et al. [34], where tTG IgA and EMA normalised in most patients within six months after liver transplantation, despite a gluten‐containing diet. The decrease in tTG IgA levels might reflect: (i) the removal of the diseased liver, which may have been the target for tTG IgA antibodies or part of a nonspecific humoral response in cirrhosis that normalised after allograft tolerance (false‐positive test); (ii) the effect of immunosuppressive drugs used to prevent allograft rejection, which may have suppressed tTGA/EMA levels, particularly in cases of true CeD (characterised by higher tTGA titers and positive EMA).

In conclusion, through systematic review and meta‐analysis, we found a prevalence of CeD in patients with PBC comparable to that of the general population. These findings do not support routine CeD screening in PBC patients unless additional CeD risk factors are present.

## Author Contributions

F.Z., C.C.: conceptualization, data curation, formal analysis, resources, visualisation, writing – original draft preparation; A.F., F.E.: methodology, writing – review and editing; N.C., I.R.: methodology, writing – original draft preparation; J.F.L.: conceptualization, project administration, supervision, writing – review and editing. All authors have commented on the manuscript and approved the final version.

## Conflicts of Interest

F.Z. has served as a speaker for Werfen, EG Stada Group, Fresenius Kabi, Kedrion, Janssen, Pfizer, Takeda, Unifarco, Malesci, Galapagos; F.Z. has served as a consultant for Galapagos, Takeda, and Tillotts Pharma. C.C.: none. A.F, has served as a speaker and advisory board member for Janssen and Tillotts Pharma. F.E. has served as an advisory board member for Boehringer Ingelheim. N.C. has served as consultant for GSK, Gilead, IPSEN, Orphalan; N.C. has served as a speaker for Orphalan, Advanz, IPSEN. I.R.: none. J.F.L. has coordinated an unrelated study on behalf of the Swedish IBD quality register (SWIBREG). That study received funding from Janssen Corporation. J.F.L. has also received financial support from Merck/MSD developing a paper reviewing national healthcare registers in China. J.F.L. has an ongoing research collaboration on celiac disease with Takeda; and about IBD with Merck/MSD.

## Supporting information


**Data S1:** liv70293‐sup‐0001‐Supinfo1.docx.

## Data Availability

All relevant data supporting the findings of this study are included in the article and in Supporting Information.

## References

[1] A. Tanaka , X. Ma , A. Takahashi , and J. M. Vierling , “Primary Biliary Cholangitis,” Lancet 404 (2024): 1053–1066. 39216494 10.1016/S0140-6736(24)01303-5

[2] F. Colapietro , A. Bertazzoni , and A. Lleo , “Contemporary Epidemiology of Primary Biliary Cholangitis,” Clinics in Liver Disease 26 (2022): 555–570. 36270716 10.1016/j.cld.2022.06.001

[3] A. F. Gulamhusein and G. M. Hirschfield , “Primary Biliary Cholangitis: Pathogenesis and Therapeutic Opportunities,” Nature Reviews. Gastroenterology & Hepatology 17 (2020): 93–110. 31819247 10.1038/s41575-019-0226-7

[4] T. Lv , S. Chen , M. Li , D. Zhang , Y. Kong , and J. Jia , “Regional Variation and Temporal Trend of Primary Biliary Cholangitis Epidemiology: A Systematic Review and Meta‐Analysis,” Journal of Gastroenterology and Hepatology 36 (2021): 1423–1434. 33141955 10.1111/jgh.15329

[5] K. D. Lindor , M. E. Gershwin , R. Poupon , M. Kaplan , N. V. Bergasa , and E. J. Heathcote , “Primary Biliary Cirrhosis,” Hepatology 50 (2009): 291–308. 19554543 10.1002/hep.22906

[6] European Association for the Study of the Liver , “EASL Clinical Practice Guidelines: The Diagnosis and Management of Patients With Primary Biliary Cholangitis,” Journal of Hepatology 67 (2017): 145–172. 28427765 10.1016/j.jhep.2017.03.022

[7] K. D. Lindor , C. L. Bowlus , J. Boyer , C. Levy , and M. Mayo , “Primary Biliary Cholangitis: 2018 Practice Guidance From the American Association for the Study of Liver Diseases,” Hepatology 69 (2019): 394–419. 30070375 10.1002/hep.30145

[8] J. F. Ludvigsson , D. A. Leffler , J. C. Bai , et al., “The Oslo Definitions for Coeliac Disease and Related Terms,” Gut 62 (2013): 43–52. 22345659 10.1136/gutjnl-2011-301346 PMC3440559

[9] P. Singh , A. Arora , T. A. Strand , et al., “Global Prevalence of Celiac Disease: Systematic Review and Meta‐Analysis,” Clinical Gastroenterology and Hepatology: The Official Clinical Practice Journal of the American Gastroenterological Association 16 (2018): 823–836.e2. 29551598 10.1016/j.cgh.2017.06.037

[10] F. Zingone , J. C. Bai , C. Cellier , and J. F. Ludvigsson , “Celiac Disease‐Related Conditions: Who to Test?,” Gastroenterology 167 (2024): 64–78. 38460606 10.1053/j.gastro.2024.02.044

[11] M. Carbone , A. Gerussi , V. Cardinale , et al., “Position Paper of the Italian Association for the Study of the Liver (AISF): Management and Treatment of Primary Biliary Cholangitis,” Digestive and Liver Disease 56 (2024): 1461–1474. 38902184 10.1016/j.dld.2024.05.002

[12] J. G. Kingham and D. R. Parker , “The Association Between Primary Biliary Cirrhosis and Coeliac Disease: A Study of Relative Prevalences,” Gut 42 (1998): 120–122. 9518232 10.1136/gut.42.1.120 PMC1726939

[13] H. T. Sorensen , A. M. Thulstrup , P. Blomqvist , et al., “Risk of Primary Biliary Liver Cirrhosis in Patients With Coeliac Disease: Danish and Swedish Cohort Data,” Gut 44 (1999): 736–738. 10205215 10.1136/gut.44.5.736 PMC1727481

[14] C. Chatzicostas , M. Roussomoustakaki , D. Drygiannakis , et al., “Primary Biliary Cirrhosis and Autoimmune Cholangitis Are Not Associated With Coeliac Disease in Crete,” BMC Gastroenterology 2 (2002): 5. 11914139 10.1186/1471-230X-2-5 PMC102761

[15] A. Lawson , J. West , G. P. Aithal , and R. F. A. Logan , “Autoimmune Cholestatic Liver Disease in People With Coeliac Disease: A Population‐Based Study of Their Association,” Alimentary Pharmacology & Therapeutics 21 (2005): 401–405. 15709990 10.1111/j.1365-2036.2005.02328.x

[16] J. F. Ludvigsson , P. Elfström , U. Broomé , et al., “Celiac Disease and Risk of Liver Disease: A General Population‐Based Study,” Clinical Gastroenterology and Hepatology: The Official Clinical Practice Journal of the American Gastroenterological Association 5 (2007): 63–69.e1. 17161656 10.1016/j.cgh.2006.09.034

[17] A. Rubio‐Tapia and J. A. Murray , “The Liver in Celiac Disease,” Hepatology 46 (2007): 1650–1658. 17969053 10.1002/hep.21949

[18] M. J. Page , J. E. McKenzie , P. M. Bossuyt , et al., “The PRISMA 2020 Statement: An Updated Guideline for Reporting Systematic Reviews,” BMJ (Clinical Research Ed.) 372 (2021): n71. 10.1136/bmj.n71 PMC8005924 33782057

[19] A. Al‐Toma , U. Volta , R. Auricchio , et al., “European Society for the Study of Coeliac Disease (ESsCD) Guideline for Coeliac Disease and Other Gluten‐Related Disorders,” United European Gastroenterol J 7, no. 5 (2019): 583–613, 10.1177/2050640619844125. PMC6545713 31210940

[20] R. van de Schoot , J. de Bruin , R. Schram , et al., “An Open Source Machine Learning Framework for Efficient and Transparent Systematic Reviews,” Nature Machine Intelligence 3 (2021): 125–133.

[21] J. Boetje and R. van de Schoot , “The SAFE Procedure: A Practical Stopping Heuristic for Active Learning‐Based Screening in Systematic Reviews and Meta‐Analyses,” Systematic Reviews 13 (2024): 81. 38429798 10.1186/s13643-024-02502-7 PMC10908130

[22] K. Sjöberg , S. Lindgren , and S. Eriksson , “Frequent Occurrence of Non‐Specific Gliadin Antibodies in Chronic Liver Disease. Endomysial but Not Gliadin Antibodies Predict Coeliac Disease in Patients With Chronic Liver Disease,” Scandinavian Journal of Gastroenterology 32 (1997): 1162–1167. 9399399 10.3109/00365529709002997

[23] W. Dickey , S. A. McMillan , and M. E. Callender , “High Prevalence of Celiac Sprue Among Patients With Primary Biliary Cirrhosis,” Journal of Clinical Gastroenterology 25 (1997): 328–329. 9412913 10.1097/00004836-199707000-00006

[24] H. M. Fidler , P. Butler , A. K. Burroughs , et al., “Co‐Screening for Primary Biliary Cirrhosis and Coeliac Disease. Primary Biliary Cirrhosis and Coeliac Disease: A Study of Relative Prevalences,” Gut 43 (1998): 300.10189863

[25] U. Volta , L. De Franceschi , N. Molinaro , et al., “Frequency and Significance of Anti‐Gliadin and Anti‐Endomysial Antibodies in Autoimmune Hepatitis,” Digestive Diseases and Sciences 43 (1998): 2190–2195. 9790453 10.1023/a:1026650118759

[26] H. R. Gillett , K. Cauch‐Dudek , E. J. L. Healthcote , et al., “Prevalence of IgA Antibodies to Endomysium and Tissue Transglutaminase in Primary Biliary Cirrhosis,” Canadian Journal of Gastroenterology and Hepatology 14 (2000): 934709. 10.1155/2000/934709 11185531

[27] U. Volta , L. Rodrigo , A. Granito , et al., “Celiac Disease in Autoimmune Cholestatic Liver Disorders,” American Journal of Gastroenterology 97 (2002): 2609–2613. 12385447 10.1111/j.1572-0241.2002.06031.x

[28] K. Kaukinen , L. Halme , P. Collin , et al., “Celiac Disease in Patients With Severe Liver Disease: Gluten‐Free Diet May Reverse Hepatic Failure,” Gastroenterology 122 (2002): 881–888. 11910339 10.1053/gast.2002.32416

[29] A. Floreani , C. Betterle , A. Baragiotta , et al., “Prevalence of Coeliac Disease in Primary Biliary Cirrhosis and of Antimitochondrial Antibodies in Adult Coeliac Disease Patients in Italy,” Digestive and Liver Disease 34 (2002): 258–261. 12038809 10.1016/s1590-8658(02)80145-1

[30] N. Bizzaro , D. Villalta , E. Tonutti , et al., “IgA and IgG Tissue Transglutaminase Antibody Prevalence and Clinical Significance in Connective Tissue Diseases, Inflammatory Bowel Disease, and Primary Biliary Cirrhosis,” Digestive Diseases and Sciences 48 (2003): 2360–2365. 14714625 10.1023/b:ddas.0000007875.72256.e8

[31] A. Habior , A. Lewartowska , J. Orłowska , et al., “Association of Coeliac Disease With Primary Biliary Cirrhosis in Poland,” European Journal of Gastroenterology & Hepatology 15 (2003): 159–164. 12560760 10.1097/00042737-200302000-00009

[32] A. E. Germenis , E. E. Yiannaki , K. Zachou , et al., “Prevalence and Clinical Significance of Immunoglobulin A Antibodies Against Tissue Transglutaminase in Patients With Diverse Chronic Liver Diseases,” Clinical and Vaccine Immunology 12 (2005): 941–948. 10.1128/CDLI.12.8.941-948.2005 PMC1182196 16085912

[33] N. Bizzaro , M. Tampoia , D. Villalta , et al., “Low Specificity of Anti‐Tissue Transglutaminase Antibodies in Patients With Primary Biliary Cirrhosis,” Journal of Clinical Laboratory Analysis 20 (2006): 184–189. 16960894 10.1002/jcla.20130 PMC6807350

[34] A. Rubio‐Tapia , A. S. Abdulkarim , R. H. Wiesner , S. B. Moore , P. K. Krause , and J. A. Murray , “Celiac Disease Autoantibodies in Severe Autoimmune Liver Disease and the Effect of Liver Transplantation,” Liver International 28 (2008): 467–476. 18339073 10.1111/j.1478-3231.2008.01681.x PMC2556252

[35] M. R. Biagini , A. Tozzi , S. Milani , et al., “Fatigue in Primary Biliary Cirrhosis: A Possible Role of Comorbidities,” European Journal of Gastroenterology & Hepatology 20 (2008): 122–126. 18188032 10.1097/MEG.0b013e3282f1cbda

[36] F. Mirzaagha , S. H. Azali , F. Islami , et al., “Coeliac Disease in Autoimmune Liver Disease: A Cross‐Sectional Study and a Systematic Review,” Digestive and Liver Disease 42 (2010): 620–623. 20236872 10.1016/j.dld.2010.02.006

[37] N. K. Gatselis , K. Zachou , G. L. Norman , et al., “IgA Antibodies Against Deamidated Gliadin Peptides in Patients With Chronic Liver Diseases,” Clinica Chimica Acta 413 (2012): 1683–1688. 10.1016/j.cca.2012.05.015 22643316

[38] J. Wakim‐Fleming , M. R. Pagadala , A. J. McCullough , et al., “Prevalence of Celiac Disease in Cirrhosis and Outcome of Cirrhosis on a Gluten Free Diet: A Prospective Study,” Journal of Hepatology 61 (2014): 558–563. 24842303 10.1016/j.jhep.2014.05.020

[39] A. Floreani , I. Franceschet , N. Cazzagon , et al., “Extrahepatic Autoimmune Conditions Associated With Primary Biliary Cirrhosis,” Clinical Reviews in Allergy and Immunology 48 (2015): 192–197. 24809534 10.1007/s12016-014-8427-x

[40] P. Muratori , A. Fabbri , C. Lalanne , M. Lenzi , and L. Muratori , “Autoimmune Liver Disease and Concomitant Extrahepatic Autoimmune Disease,” European Journal of Gastroenterology & Hepatology 27 (2015): 1175–1179. 26148248 10.1097/MEG.0000000000000424

[41] K. Callichurn , L. Cvetkovic , A. Therrien , C. Vincent , P. O. Hétu , and M. Bouin , “Prevalence of Celiac Disease in Patients With Primary Biliary Cholangitis,” Journal of the Canadian Association of Gastroenterology 4 (2021): 44–47. 33644676 10.1093/jcag/gwz039 PMC7898370

[42] C. Efe , M. Torgutalp , I. Henriksson , et al., “Extrahepatic Autoimmune Diseases in Primary Biliary Cholangitis: Prevalence and Significance for Clinical Presentation and Disease Outcome,” Journal of Gastroenterology and Hepatology 36 (2021): 936–942. 32790935 10.1111/jgh.15214

[43] E. Yehezkel , I. Israel , I. Houri , M. Leshno , O. Shibolet , and E. Zigmond , “Real‐World Management of Patients With Primary Biliary Cholangitis‐A Retrospective Study From a Tertiary Medical Center in Israel,” Journal of Clinical Medicine 10 (2021): 4551. 34640567 10.3390/jcm10194551 PMC8509713

[44] A. A. Hitawala , A. Almomani , S. Onwuzo , A. Boustany , P. Kumar , and I. Asaad , “Prevalence of Autoimmune, Cholestatic and Nonalcoholic Fatty Liver Disease in Celiac Disease,” European Journal of Gastroenterology & Hepatology 35 (2023): 1030–1036. 37395201 10.1097/MEG.0000000000002599

[45] Z. Munn , S. Moola , K. Lisy , D. Riitano , and C. Tufanaru , “Methodological Guidance for Systematic Reviews of Observational Epidemiological Studies Reporting Prevalence and Cumulative Incidence Data,” International Journal of Evidence‐Based Healthcare 13 (2015): 147–153. 26317388 10.1097/XEB.0000000000000054

[46] V. N. Nyaga , M. Arbyn , and M. Aerts , “Metaprop: A Stata Command to Perform Meta‐Analysis of Binomial Data,” Archives of Public Health 72 (2014): 39. 25810908 10.1186/2049-3258-72-39 PMC4373114

[47] A. Sood , M. S. Khurana , R. Mahajan , et al., “Prevalence and Clinical Significance of IgA Anti‐Tissue Transglutaminase Antibodies in Patients With Chronic Liver Disease,” Journal of Gastroenterology and Hepatology 32 (2017): 446–450. 27346589 10.1111/jgh.13474

[48] D. Villalta , M. Crovatto , S. Stella , E. Tonutti , R. Tozzoli , and N. Bizzaro , “False Positive Reactions for IgA and IgG Anti‐Tissue Transglutaminase Antibodies in Liver Cirrhosis Are Common and Method‐Dependent,” Clinica Chimica Acta 356 (2005): 102–109. 10.1016/j.cccn.2005.01.015 15936306

[49] D. A. Leffler and D. Schuppan , “Update on Serologic Testing in Celiac Disease,” American Journal of Gastroenterology 105 (2010): 2520–2524. 21131921 10.1038/ajg.2010.276

[50] F. Zingone , G. L. Norman , E. Smecuol , et al., “Utilizing Both IgA Tissue Transglutaminase and IgG‐Deamidated Gliadin Peptide Antibodies Offers Accurate Celiac Disease Diagnosis Without Duodenal Biopsy,” Digestive and Liver Disease 57 (2025): 609–615. 39472176 10.1016/j.dld.2024.10.010

[51] C. Ciacci , J. C. Bai , G. Holmes , et al., “Serum Anti‐Tissue Transglutaminase IgA and Prediction of Duodenal Villous Atrophy in Adults With Suspected Coeliac Disease Without IgA Deficiency (Bi.A.CeD): A Multicentre, Prospective Cohort Study,” Lancet Gastroenterology & Hepatology 8 (2023): 1005–1014. 37696284 10.1016/S2468-1253(23)00205-4

