# Supplementary material for: Prevalence of Celiac Disease in Patients With Primary Biliary Cholangitis: A Systematic Review and Meta‐Analysis
Source: Liver Int. 2025 Aug 19;45(9):e70293. doi: 10.1111/liv.70293 (PMC12363381; doi:10.1111/liv.70293)
Supplement: Supplementary file 1 — Data S1: liv70293‐sup‐0001‐Supinfo1.docx. [file LIV-45-0-s001.docx]

**SUPPLEMENTARY**

**Suppl. Table S1:** Complete search strategy

. Medline

| Interface: Ovid MEDLINE(R) ALL  Date of Search: 2024-03-12  Number of hits: 652  Comment: In Ovid, two or more words are automatically searched as phrases; i.e. no quotation marks are needed | Field labels   - exp/ = exploded MeSH term - / = non exploded MeSH term - .ti,ab,kf. = title, abstract and author keywords - adjx = within x words, regardless of order - * = truncation of word for alternate endings |
| --- | --- |
| Ovid MEDLINE(R) ALL <1946 to March 11, 2024>   \| 1 \| Celiac Disease/ \| 22184 \| \| --- \| --- \| --- \| \| 2 \| exp Glutens/ \| 9944 \| \| 3 \| exp Transglutaminases/ \| 7828 \| \| 4 \| (celiac* or celiak* or coeliac* or coeliak* or 'non-tropical sprue' or 'nontropical sprue').ti,ab,kf. \| 33018 \| \| 5 \| (Gluten* or gliadin* or wheat or antigliadin or aga).ab,kf,ti. \| 95377 \| \| 6 \| (endomys* or antiendomys* or ema or aea or transglutamin* or anti-transglutamin* or trans glutamin* or ttg or tta or tgm2 or tgase or Factor XIII or Factor 13A or Factor Thirteen A).ti,ab,kf. \| 33339 \| \| 7 \| ((villus or villous) adj3 atroph*).ti,ab,kf. \| 2883 \| \| 8 \| or/1-7 \| 154622 \| \| 9 \| Liver Cirrhosis, Biliary/ \| 8867 \| \| 10 \| Liver Cirrhosis/ \| 87466 \| \| 11 \| ((C?olesta* or autoimmune) adj3 liver).ti,ab,kf. \| 8346 \| \| 12 \| ((biliary or hanot or hepatic or hypertrophic or liver) adj3 (c?olangi* or cirr?hos* or fibros*)).ti,ab,kf. \| 99777 \| \| 13 \| (Anti mitochondrial antibod* or antimitochondri* antibod* or mitochondri* antibod* or PBC).ti,ab,kf. \| 7831 \| \| 14 \| 9 or 10 or 11 or 12 or 13 \| 150439 \| \| 15 \| 14 and 8 \| 881 \| \| 16 \| limit 15 to yr="1990 -Current" \| 652 \| | |

2. Embase

| Interface: embase.com  Date of Search: 2024-03-12  Number of hits: 1666  Comment: Emtree is the controlled vocabulary in Embase | Field labels   - /exp = exploded Emtree term - /de = non exploded Emtree term - ti,ab,kw = title, abstract and author keywords - NEAR/x = within x words, regardless of order - * = truncation of word for alternate endings |
| --- | --- |
| \| No. \| Query \| Results \| \| --- \| --- \| --- \| \| #22 \| #19 NOT #20 AND [1990-2024]/py \| 1666 \| \| #21 \| #19 NOT #20 \| 1900 \| \| #20 \| #12 AND #18 AND ([conference abstract]/lim OR [conference paper]/lim OR [conference review]/lim) \| 659 \| \| #19 \| #12 AND #18 \| 2072 \| \| #18 \| #13 OR #14 OR #15 OR #16 OR #17 \| 275523 \| \| #17 \| 'anti mitochondri* antibod*':ti,ab,kw OR 'antimitochondri* antibod*':ti,ab,kw OR 'mitochondri* antibod*':ti,ab,kw OR pbc:ti,ab,kw \| 13107 \| \| #16 \| ((biliary OR hanot OR hepatic OR hypertrophic OR liver) NEAR/3 (c$olangi* OR cirr$hos* OR fibros*)):ti,ab,kw \| 153494 \| \| #15 \| ((c$olesta* OR autoimmune) NEAR/3 'liver'):ti,ab,kw \| 14138 \| \| #14 \| 'liver cirrhosis'/de \| 176686 \| \| #13 \| 'biliary cirrhosis'/de OR 'primary biliary cirrhosis'/de \| 19111 \| \| #12 \| #1 OR #2 OR #3 OR #4 OR #5 OR #6 OR #7 OR #8 OR #9 OR #10 OR #11 \| 197819 \| \| #11 \| ((villus OR villous) NEAR/3 atroph*):ti,ab,kw \| 4457 \| \| #10 \| endomys*:ti,ab,kw OR antiendomys*:ti,ab,kw OR ema:ti,ab,kw OR aea:ti,ab,kw OR transglutamin*:ti,ab,kw OR 'anti transglutamin*':ti,ab,kw OR 'trans glutamin*':ti,ab,kw OR ttg:ti,ab,kw OR tta:ti,ab,kw OR tgm2:ti,ab,kw OR tgase:ti,ab,kw OR 'factor xiii':ti,ab,kw OR 'factor 13a':ti,ab,kw OR 'factor thirteen a':ti,ab,kw \| 49977 \| \| #9 \| gluten*:ti,ab,kw OR gliadin*:ti,ab,kw OR wheat:ti,ab,kw OR antigliadin:ti,ab,kw OR aga:ti,ab,kw \| 108322 \| \| #8 \| celiac*:ti,ab,kw OR celiak*:ti,ab,kw OR coeliac*:ti,ab,kw OR coeliak*:ti,ab,kw OR 'non-tropical sprue':ti,ab,kw OR 'nontropical sprue':ti,ab,kw \| 50226 \| \| #7 \| 'endomysium antibody'/de \| 2712 \| \| #6 \| 'endomysium'/de \| 654 \| \| #5 \| 'protein glutamine gamma glutamyltransferase 2'/de \| 3401 \| \| #4 \| 'protein glutamine gamma glutamyltransferase'/de \| 9058 \| \| #3 \| 'gliadin'/de \| 4644 \| \| #2 \| 'gluten'/de \| 11900 \| \| #1 \| 'celiac disease'/de \| 40896 \| | |

3. Web of Science Core Collection

| Interface: Clarivate Analytics  Editions = A&HCI , ESCI , SCI-EXPANDED , SSCI  Date of Search: 2024-03-12  Number of hits: 741 | Field labels   - TS/Topic = title, abstract, author keywords and Keywords Plus - NEAR/x = within x words, regardless of order - * = truncation of word for alternate endings   Note: the *Exact search*-function was used for all the searches |
| --- | --- |
| \| # \| Search Query \| Results \| \| --- \| --- \| --- \| \| 1 \| TS=(celiac* OR celiak* OR coeliac* OR coeliak* OR "non-tropical sprue" OR "nontropical sprue" ) \| 38585 \| \| 2 \| TS=(Gluten* OR gliadin* OR wheat OR antigliadin OR aga ) \| 275309 \| \| 3 \| TS=(endomys* OR antiendomys* OR ema OR aea OR transglutamin* OR anti-transglutamin* OR "trans glutamin*" OR ttg OR tta OR tgm2 OR tgase OR "Factor XIII" OR "Factor 13A" OR "Factor Thirteen A" ) \| 45830 \| \| 4 \| TS=((villus OR villous ) NEAR/3 atroph* ) \| 2827 \| \| 5 \| #4 OR #3 OR #2 OR #1 \| 341802 \| \| 6 \| TS=((C$olesta* OR autoimmune ) NEAR/3 liver ) \| 9629 \| \| 7 \| TS=((biliary OR hanot OR hepatic OR hypertrophic OR liver ) NEAR/3 (c$olangi* OR cirr$hos* OR fibros* )) \| 109476 \| \| 8 \| TS=("Anti mitochondrial antibod*" OR "antimitochondri* antibod*" OR "mitochondri* antibod*" OR PBC ) \| 10314 \| \| 9 \| #6 OR #7 OR #8 \| 120448 \| \| 10 \| #5 AND #9 \| 756 \| \| 11 \| #5 AND #9 Timespan: 1990-01-01 to 2024-12-31 \| 741 \| | |

4. Cochrane Library

| Interface: Wiley  Date of Search: 2024-03-12  Number of hits: 45 | Field labels   - ti,ab,kw = title, abstract and author keywords - NEAR/x = within x words, regardless of order - * = truncation of word for alternate endings |
| --- | --- |
| \| ID \| Search \| Hits \| \| --- \| --- \| --- \| \| #1 \| [mh ^"Celiac Disease"] \| 490 \| \| #2 \| [mh Glutens] \| 228 \| \| #3 \| [mh Transglutaminases] \| 67 \| \| #4 \| (celiac*:ti,ab,kw OR celiak*:ti,ab,kw OR coeliac*:ti,ab,kw OR coeliak*:ti,ab,kw OR "'non-tropical sprue'":ti,ab,kw OR "'nontropical sprue'":ti,ab,kw) \| 1566 \| \| #5 \| (Gluten*:ti,ab,kw OR gliadin*:ti,ab,kw OR wheat:ti,ab,kw OR antigliadin:ti,ab,kw OR aga:ti,ab,kw) \| 3966 \| \| #6 \| (endomys*:ti,ab,kw OR antiendomys*:ti,ab,kw OR ema:ti,ab,kw OR aea:ti,ab,kw OR transglutamin*:ti,ab,kw OR anti-transglutamin*:ti,ab,kw OR ("trans" NEXT glutamin*):ti,ab,kw OR ttg:ti,ab,kw OR tta:ti,ab,kw OR tgm2:ti,ab,kw OR tgase:ti,ab,kw OR "Factor XIII":ti,ab,kw OR "Factor 13A":ti,ab,kw OR "Factor Thirteen A":ti,ab,kw) \| 2104 \| \| #7 \| ((villus:ti,ab,kw OR villous:ti,ab,kw) NEAR/3 atroph*:ti,ab,kw) \| 75 \| \| #8 \| #1 OR #2 OR #3 OR #4 OR #5 OR #6 OR #7 \| 6774 \| \| #9 \| [mh ^"Liver Cirrhosis, Biliary"] \| 387 \| \| #10 \| [mh ^"Liver Cirrhosis"] \| 3458 \| \| #11 \| ((C?olesta*:ti,ab,kw OR autoimmune:ti,ab,kw) NEAR/3 ("liver"):ti,ab,kw) \| 429 \| \| #12 \| ((biliary:ti,ab,kw OR hanot:ti,ab,kw OR hepatic:ti,ab,kw OR hypertrophic:ti,ab,kw OR liver:ti,ab,kw) NEAR/3 (c?olangi*:ti,ab,kw OR cirr?hos*:ti,ab,kw OR fibros*:ti,ab,kw)) \| 11318 \| \| #13 \| (("Anti mitochondrial" NEXT antibod*):ti,ab,kw OR (antimitochondri* NEXT antibod*):ti,ab,kw OR (mitochondri* NEXT antibod*):ti,ab,kw OR PBC:ti,ab,kw) \| 850 \| \| #14 \| #9 OR #10 OR #11 OR #12 OR #13 \| 11803 \| \| #15 \| #14 AND #8 with Publication Year from 1990 to 2024, with Cochrane Library publication date Between Jan 1990 and Dec 2024, in Trials \| 45 \| | |

**Suppl. Table S2:** Quality assessment of included studies using JBI Critical Appraisal Checklist for included Prevalence Studies

|  | **Domains** | | | | | | | | |
| --- | --- | --- | --- | --- | --- | --- | --- | --- | --- |
|  | **1** | **2** | **3** | **4** | **5** | **6** | **7** | **8** | **9** |
| **Study** | Was the sample frame appropriate to address the target population? | Were study participants sampled in an appropriate way? | Was the sample size adequate? | Were the study subjects and the setting described in detail? | Was the data analysis conducted with sufficient coverage of the identified sample? | Were valid methods used for the identification of the condition? | Was the condition measured in a standard, reliable way for all participants? | Was there appropriate statistical analysis? | Was the response rate adequate, and if not, was the low response rate managed appropriately? |
| **Sjoberg 1997** | Yes | NA | 1 | yes |  | Yes | yes | yes | NA |
| **Dickey 1997** | Yes | NA | 0 | yes |  | Yes | no (2/6 no bio) | yes | NA |
| **Findler, 1998** | yes (letter to the editor) | NA | 0 | no |  | Yes | yes | yes | NA |
| **Volta 1998** | yes (serum) | NA | 0 | yes |  | Yes | yes | yes | NA |
| **Kingham 1998** | Yes | NA | 0 | no |  | No (databases based on guidelines) | yes | yes | NA |
| **Gillett 2000** | ? Stored sera from 378 patients with PBC from The Toronto Western Hospital, Toronto, Ontario, were screened | NA | 1 | yes |  | Yes | no (5/10 no bio) | yes | NA |
| **Volta, 2002** | Yes | NA | 1 | yes |  | Yes | yes | yes | NA |
| **Chatzicostas 2002** | Yes | NA | 0 | yes |  | Yes | no (7/17 no bio) | yes | NA |
| **Kaukinen 2002** | no (underwent OLT) | NA | 0 | no |  | Yes | yes | yes | NA |
| **Floreani 2002** | Yes | NA | 0 | yes |  | Yes | no (4/24 no bio) | yes | NA |
| **Bizzarro 2003** | Yes | NA | 0 | No |  | Yes | yes | yes | NA |
| **Habior 2003** | yes?: Serum samples collected from 115 consecutive patients with PBC (108 women, 7 men; median age 55 years, range 28–78 years) who had been diagnosed and followed up in the period 1996–2002 at the Department of Gastroenterology | NA | 1 | yes |  | Yes | no (3/8 no bio) | yes | NA |
| **Germenis 2005** | Yes | NA | 0 | yes |  | Yes | no (unknown) | yes | NA |
| **Bizzarro 2006** | Yes | NA | 1 | yes |  | Yes | no (6/28 no bio) | yes | NA |
| **Tabia 2007** | no (patients underwent olt) | NA | 1 | No |  | No | no (2/2 no bio) | yes | NA |
| **Biagini 2008** | no (only patients underwent fatigue test) | NA | 0 | yes |  | no (patients history, table 1) |  | yes | NA |
| **Mirzaagha 2010** | Yes | NA | 0 | yes |  | Yes | no (1/1 no bio) | yes | NA |
| **Gatselis 2012** | Yes | NA | 0 | yes |  | Yes | yes | yes | NA |
| **Wakim Fleming 2014** | no (patients with biopsy proven cirrhosis scheduled for an upper endoscopy) | NA | 0 | No |  | yes | yes | yes | NA |
| **Floreani 2015** | Yes | NA | 1 | yes |  | no (patients history) | yes | yes | NA |
| **Muratori 2015** | Yes | NA | 1 | yes |  | no (international criteria) | yes | yes | NA |
| **Callichurn 2021** | no (patients underwent ttg) | NA | 0 | No |  | no (patients history) | yes | yes | NA |
| **Efe 2021** | Si | NA | 1 | yes |  | no (retrived from database) | yes | yes | NA |
| **Yehezkel 2021** | Si | NA | 1 | yes |  | No | yes | yes | NA |
| **Hitawala, 2023** | Si | NA | 1 | yes |  | no (retrived from database) | yes | yes | NA |

**P=0.558**

**Suppl. Fig S1: Meta-regression analysis of the prevalence of coeliac disease in patients with PBC by publication year (n=22)**

**P=0.734**

**Supp Fig S2: Meta-regression analysis of the prevalence of coeliac disease in patients with PBC by sample size (n=22)**

**P= 0.693**

**Supp Fig S3: Meta-regression analysis of the prevalence of coeliac disease in patients with PBC by proportion females (n=17)**

**P= 0.323**

**Supp Fig S4: Meta-regression analysis of the prevalence of coeliac disease in patients with PBC by mean age of the study population (n=13)**

**Supp Fig. S5. Prevalence of serology-confirmed (IgA EMA + IgA tTG) coeliac disease in PBC (n=11)**
